# Improving Nursing Team Collaboration Through Nurses’ Digital Literacy: A Variable‐Centered and Person‐Centered Perspective

**DOI:** 10.1155/jonm/7654712

**Published:** 2025-12-11

**Authors:** Xiaoping Zhang, Zhongcheng An, Haifang Zhou, Bing Wu, Lumeng Lu

**Affiliations:** ^1^ Department of Geriatric Medicine, The Second Affiliated Hospital of Zhejiang Chinese Medical University, Hangzhou, 310007, Zhejiang, China, z2hospital.com; ^2^ Department of Orthopedics, The Second Affiliated Hospital of Zhejiang Chinese Medical University, Hangzhou, Zhejiang, China, z2hospital.com; ^3^ Department of Nursing, Hangzhou TCM Hospital Affiliated to Zhejiang Chinese Medical University, Hangzhou, 310007, Zhejiang, China, hztcm.net; ^4^ The Second Clinical College, Zhejiang Chinese Medical University, Hangzhou, China, zcmu.edu.cn

**Keywords:** digital literacy, nurses, nursing team collaboration ability, psychological safety climate, situational awareness

## Abstract

**Background:**

Digital technologies have transformed nursing practices, enhancing efficiency while increasing the complexity of information. Although digital literacy enables nurses to manage digital tools effectively, its impact on team collaboration remains underexplored. This study investigates the influence of digital literacy on nursing team collaboration ability and its underlying mechanisms.

**Methods:**

This study was conducted from March to July 2025 in a tertiary Grade A hospital in Hangzhou, China, involving 495 nurses. We employed scales for digital literacy, situational awareness, psychological safety climate, and team collaboration to collect data. The Process Model 6 was used to test the chain‐like mediating effects of situational awareness and psychological safety climate on the relationship between nurses’ digital literacy and their ability to collaborate within the nursing team. Latent profile analysis was applied to classify situational awareness and psychological safety climate into latent categories.

**Results:**

Digital literacy is significantly and positively associated with nursing team collaboration. Further mediation analysis revealed that situational awareness and psychological safety climate acted as chain‐like mediators between digital literacy and team collaboration. From the individual‐centered perspective, situational awareness and psychological safety climate were categorized into three subgroups: high psychological safety climate‐high situational awareness, medium psychological safety climate‐medium situational awareness, and low psychological safety climate‐low situational awareness.

**Conclusion:**

Hospital administrators and nursing team leaders should prioritize the development of nurses’ digital literacy and optimize the situational awareness and psychological safety climate in nursing environments to enhance team collaboration and overall healthcare service quality.

## 1. Introduction

In the era of digitalized healthcare, the deep integration of information technology and intelligent tools has significantly improved the accuracy and accessibility of nursing services [1–3]. For instance, the application of AI‐driven diagnostics, wearable sensors, and cross‐language virtual assistants is driving healthcare towards a continuous, preventive [4, 5], and highly accessible model, extending nursing care into community and home settings [6–8]. However, this increasing reliance on technology has intensified the digital adaptation pressure on clinical nurses, defined as the cognitive overload and anxiety stemming from rapid technological advancements and the digitization of workflows [9–12]. For example, Lee and Lee [13] found that 46% to 64% of nurses reported frequently managing updates to electronic health records (EHRs), operating remote monitoring platforms, and handling multidevice coordination tasks, with the complexity of these technologies often exceeding their current digital literacy. Consequently, recent research has focused on enhancing the digital literacy of clinical nurses [14–16]. Digital literacy refers to an individual’s ability to acquire, understand, and apply digital technologies effectively. Existing research has primarily concentrated on issues such as information anxiety [17], digital proficiency [18], and digital health knowledge [19]. For example, Kwon and Oh [20] demonstrated a significant positive correlation between nurses’ digital literacy and health‐promoting behaviors. However, this study did not explore the relationship between digital literacy and team collaboration. This omission is unfortunate, as digital literacy enables nurses to operate tools such as EHRs and telemedicine systems proficiently, facilitating real‐time data updates and cross‐departmental information sharing [21, 22]. For instance, through EHR systems, nursing teams can simultaneously access patient vital signs and medication records, reducing communication delays and errors [23]. Thus, examining the relationship between clinical nurses’ digital literacy and nursing team collaboration is of significant clinical importance.

Nursing team collaboration refers to the ability of nursing professionals to exchange professional knowledge, complement skills, provide emotional support, and collectively solve problems to maximize team effectiveness in healthcare institutions [24, 25]. Its core lies in goal alignment and action coordination [26], with key characteristics including goal‐driven orientation, cross‐professional collaboration, shared responsibility, mutual trust, and dynamic adaptability [27]. These features and advantages have gradually become a new research hotspot in the field of nursing. For example, Dun [28] explored how critical thinking skills among nurses positively influence nursing team collaboration. Prior studies have analyzed factors such as leadership, role allocation, team culture and values, information sharing, and communication to understand how to improve nursing team collaboration [25, 29–32]. For instance, Sharma and Sharma [33] investigated how digital technologies, such as ChatGPT and the Metaverse, could enhance collaboration within nursing teams. These studies indicate that modern technologies have provided new momentum for improving the efficiency of nursing team collaboration [34, 35]. In this process, nurses’ digital literacy plays a key role. For example, nurses can share patient case information in real‐time through online systems and communicate with patients and their families via video conferencing, thereby enhancing the speed, accuracy, and efficiency of information transmission and team collaboration [36, 37]. However, few prior studies have analyzed the relationship between nurses’ digital literacy and team collaboration. Therefore, this study attempts to address three key questions: (1) Does nurses’ digital literacy have a positive impact on nursing team collaboration? (2) Do situational awareness and psychological safety climate act as chain‐like mediators in this relationship? (3) Do situational awareness and psychological safety climate exhibit heterogeneity among nurses?

### 1.1. Variable‐Centered Perspective

#### 1.1.1. Psychological Safety Climate

Psychological safety climate refers to the shared belief that individuals can learn from risks and uncertainties, emphasizing its importance for team learning behaviors and innovation in healthcare settings [38, 39]. Extensive research has introduced this concept into healthcare and nursing, linking it to team performance and patient safety [40, 41]. For example, nurses working in environments with higher psychological safety demonstrate better job performance, including enhanced decision‐making skills, higher job satisfaction, and reduced psychological stress [42, 43]. Prior studies have consistently shown that psychological safety climate is closely associated with mature team collaboration, open communication, and supportive team environments [44]. However, previous research has not explored the relationship between psychological safety climate and digital literacy. With the increasing adoption of modern medical technologies, more researchers are attempting to validate the relationship between nurses’ digital literacy and psychological safety climate, as this is crucial for enhancing nurses’ adaptability in digitalized healthcare environments. In teams with high psychological safety, nurses are more willing to experiment with new technologies and integrate them into daily practice [45]. They are also more inclined to seek help and collaborate when encountering technical issues. Such an environment not only promotes individual digital literacy development but also provides a stronger foundation for nursing team collaboration.

Further analysis reveals that psychological safety climate may influence clinical nurses’ digital literacy and team collaboration through multiple pathways. On the one hand, a psychological safety climate fosters open communication and knowledge sharing, which in turn impacts nurses’ acceptance and willingness to use digital tools [46, 47]. In safe environments, nurses are more likely to share experiences and learning opportunities with new technologies, thereby accelerating their individual digital literacy development [48, 49]. On the other hand, a psychological safety climate reduces defensive thinking among nurses, making them more open to learning and experimenting with new technologies [50]. This further strengthens the accumulation of digital literacy.

Regarding nursing team collaboration, psychological safety climate enhances mutual trust and a sense of belonging among team members by providing emotional support and avoiding a blame culture [51]. When facing technical issues or work conflicts, nurses are more likely to seek solutions through collaboration [52]. This not only improves the team’s overall adaptability to new technologies but also optimizes collaborative patterns. Therefore, the psychological safety climate acts as a bridge between organizational culture and individual behavior, mediating the relationship between digital literacy and team collaboration by influencing information‐sharing intentions and team trust.

Based on the above analysis, this study proposes the following hypotheses:

• H1: Nurses’ digital literacy has a positive impact on nursing team collaboration.
• H2: Psychological safety climate mediates the relationship between nurses’ digital literacy and nursing team collaboration.

#### 1.1.2. Situational Awareness

Situational awareness refers to an individual’s ability to perceive and understand their environment within a specific context, emphasizing the deep interaction between cognitive activities and situational factors [53, 54]. In nursing research, situational awareness has been increasingly recognized as a critical ability for clinical nurses to adapt and make informed decisions in complex work environments [55, 56]. The core dimensions of situational awareness include environmental perception, temporal perception, and social perception [57]. In nursing practice, these dimensions are dynamically perceived and integrated through multiple sensory channels, such as visual, auditory, and tactile inputs. For example, Weigl et al. [58] demonstrated through empirical research that high levels of situational awareness significantly improve nurses’ ability to quickly identify medical device malfunctions and optimize the efficiency of nursing workflows. However, prior research has not sufficiently explored how situational awareness influences nurses’ team collaboration in the context of digitalized healthcare environments.

To address this gap, this study posits that situational awareness enhances nurses’ familiarity with digital tools in their environment, thereby indirectly promoting the development of digital literacy. According to Bandura and Wessels [59], social cognitive theory suggests that individual behavior and ability development are deeply influenced by environmental feedback. In complex clinical environments, nurses with high situational awareness can more quickly and accurately identify the functions and operational requirements of digital devices, thereby enhancing their confidence and ability to use digital tools. This capability extends beyond technical operation to include the development of digital information analysis and decision‐making skills.

Situational awareness also optimizes team collaboration by enhancing nurses’ ability to perceive collaborative contexts dynamically. The development of nursing team collaboration depends on team members’ clear understanding of their roles and task distributions [59, 60]. According to team collaboration theory [61], situational awareness enables nurses to understand potential conflict points better and the collaboration needs within the team, facilitating more effective information sharing and coordinated actions during nursing practice. For example, in high‐pressure environments such as emergency rooms, nurses with high situational awareness can rapidly identify the workload and resource allocation needs of team members, thereby optimizing the configuration of nursing resources and improving overall team collaboration efficiency [62]. Moreover, situational awareness enhances nurses’ ability to keenly perceive patient needs, significantly improving the quality of care as perceived by patients.

Based on the above analysis, this study proposes the following hypothesis:

• H3: Situational awareness mediates the relationship between nurses’ digital literacy and nursing team collaboration.

#### 1.1.3. Chain‐Like Mediating Role of Psychological Safety Climate and Situational Awareness

Drawing from social cognitive theory and team effectiveness frameworks [62], psychological safety climate serves as a social cognitive premise for transforming digital literacy into effective situational awareness in digitalized nursing contexts. In such environments, high psychological safety reduces anxiety related to technology use, encouraging nurses to explore advanced features of EHR systems and clinical decision‐support systems and minimizing information avoidance behaviors caused by fear of technological errors [63, 64]. Simultaneously, psychological safety climate fosters knowledge sharing, as nurses are more willing to disclose technical operation challenges and data interpretation concerns, allowing them to address individual digital skill gaps through peer learning [65]. Self‐efficacy theory further explains that psychological safety enhances efficiency beliefs in technology use [66], motivating nurses to proactively analyze and predict risks from multiple data sources rather than mechanically executing operational procedures.

The high‐quality situational awareness fostered by a psychological safety climate further enhances nursing team collaboration. Nurses with high situational awareness can precisely communicate predictions of patient conditions, enabling the team to form shared mental models [67]. Additionally, psychological safety ensures that nurses have the authority to provide real‐time feedback, guaranteeing that situational awareness information is promptly calibrated [68]. According to Salas’ team collaboration model, this closed‐loop communication based on accurate situational awareness reduces coordination delays, particularly in multitasking emergency scenarios [69]. Thus, digital literacy, supported by psychological safety climate, deepens situational awareness, and situational awareness, as a cognitive hub, translates individual digital capabilities into coordinated team actions.

Based on the above analysis, this study proposes the following hypothesis:

• H4: Psychological safety climate and situational awareness act as chain‐like mediators between nurses’ digital literacy and nursing team collaboration.

### 1.2. Personal‐Centered Perspective

Recent studies have revealed significant discrepancies in the relationship between psychological safety climate and situational awareness [70, 71]. Some research indicates a strong positive correlation between the two, suggesting that a favorable psychological safety climate can significantly enhance nursing staff’s situational awareness, thereby optimizing nursing decisions and team collaboration [72, 73]. However, other studies have found no significant relationship between the two, and in some cases, even a negative correlation, positing that an overemphasis on psychological safety climate may lead to a decline in nursing staff’s ability to perceive complex situations [74, 75]. These contradictory research findings may stem from the variable‐centered approach adopted in study designs, where psychological safety climate and situational awareness are treated as independent, static variables. This approach may overlook the dynamic interplay between the two constructs, potentially masking their deeper connections and resulting in inconsistent findings.

We observed that the measurement tools and operational definitions of psychological safety climate and situational awareness vary significantly across different studies. For instance, some studies employ self‐report scales to measure psychological safety climate, while others use observational methods or peer evaluations [76]. Such methodological differences may contribute to discrepancies in the results. Additionally, the specific contexts and backgrounds focused on in these studies differ. For example, some research targets high‐intensity environments, such as emergency departments, while others focus on general wards, potentially neglecting contextual differences [77, 78]. Consequently, the inconsistencies in existing research may arise from an incomplete understanding of the complex relationship between psychological safety climate and situational awareness.

To address these issues, this study employs latent profile analysis to uncover the underlying causal mechanisms and intrinsic logical relationships between psychological safety climate and situational awareness. Unlike traditional variable‐centered analysis, latent profile analysis is better equipped to capture dynamic relationships within complex systems [79], revealing how psychological safety climate influences the formation and development of situational awareness through multiple pathways. This method not only handles nonlinear relationships between variables but also reveals differences in the relationship between psychological safety climate and situational awareness across various contexts [80]. Through latent profile analysis, this study aims to provide a more comprehensive theoretical framework for the field of nursing, guiding hospital administrators and nursing team leaders in optimizing the psychological safety climate, enhancing nursing staff’s situational awareness, and ultimately improving the quality of care and patient safety.

### 1.3. The Present Study

**Figure 1:**
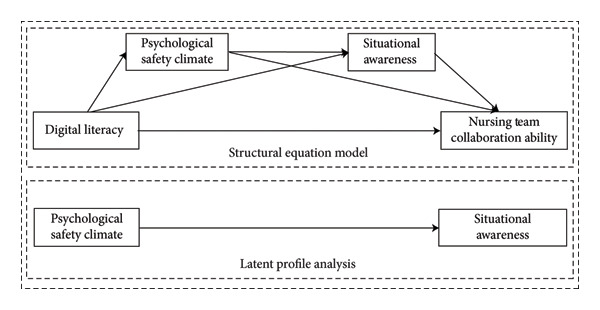
Theoretical framework model.

This study examines the impact of nurses’ digital literacy on nursing team collaboration ability from a variable‐centered perspective, as well as the internal mechanisms underlying this relationship. From a variable‐centered perspective, we analyzed the heterogeneity of nurses’ psychological safety climate and situational awareness. The theoretical framework model is illustrated in Figure 1.

## 2. Methods

### 2.1. Participants

#### 2.1.1. Recruitment of Participants

This study was approved by the Ethics Committee of the Affiliated Hangzhou Hospital of Zhejiang Chinese Medical University, allowing the conduct of this cross‐sectional study. The sample was recruited between March and July 2025 at a public tertiary Grade A hospital with approximately 1500 open beds, about 2000 fixed beds, and about 40 clinical and medical technology departments in Hangzhou, China. This setting represents a large‐scale, government‐funded institution specializing in integrated traditional Chinese and Western medicine, providing a typical context for urban nursing practices in eastern China. We contacted the director of the hospital’s nursing department, informed them of the research purpose, procedures, and risks, and obtained their consent before commencing data collection. Participants completed the data collection via mobile electronic devices.

Concurrently, all participants were required to sign an informed consent form before completing the online questionnaire. The informed consent information was presented at the beginning of the online survey, detailing the study’s purpose, procedures, potential risks or benefits, and the voluntary nature of participation. Only participants who selected the option indicating their consent were allowed to proceed with the online questionnaire. Completing all the required questions took approximately 5 min. The study obtained informed consent from all participants.

#### 2.1.2. Inclusion and Exclusion Criteria

Inclusion criteria: (1) Participants were clinical nurses with at least 1 year of clinical nursing experience. (2) Participants worked in a tertiary Grade A hospital. (3) Participants had used digital tools daily for at least 3 months to ensure basic digital literacy. (4) Participants voluntarily signed the informed consent form, explicitly agreeing to participate in the study after being informed of its purpose, data usage, and privacy protection measures. (5) Participants were proficient in Chinese and had no significant cognitive impairments.

Exclusion criteria: (1) Exclusion of nurse managers, nursing department directors, nursing educators, and other non‐direct‐care nursing staff. (2) Exclusion of contract nurses, temporary workers, or part‐time nurses. (3) Exclusion of nurses who rarely use digital tools. (4) Exclusion of nurses who could not understand Chinese or were non‐native. (5) Exclusion of participants who had recently experienced significant life events. (6) Exclusion of participants with mental health conditions.

#### 2.1.3. Demographic Information

**Figure 2:**
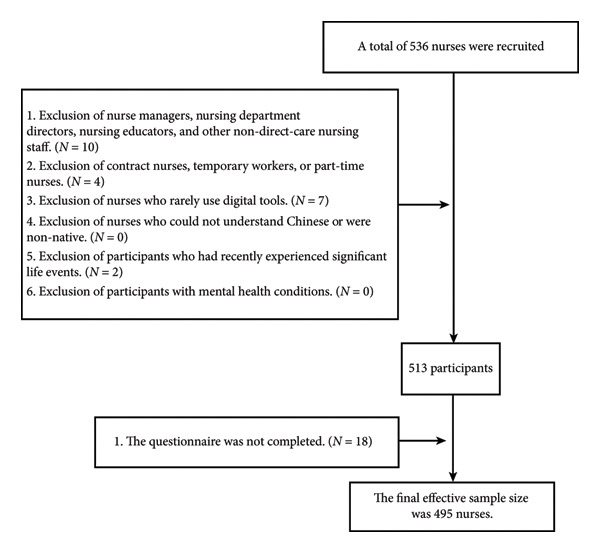
Data cleaning process.

A total of 536 participants were recruited. After applying the inclusion and exclusion criteria, 23 participants were excluded, leaving 513 eligible participants. Subsequently, 18 incomplete questionnaires were removed, resulting in 495 valid questionnaires and yielding an effective response rate of 92.35%. The specific data cleaning process is shown in Figure 2. Among the participants, 84 (17.0%) were male nurses, and 411 (83.0%) were female. The age distribution included 154 nurses (31.1%) aged 18–30 years, 113 nurses (22.8%) aged 31–45 years, 169 nurses (34.1%) aged 46–60 years, and 59 nurses (11.9%) aged 61 years and older. In terms of education, 84 nurses (17.0%) held an associate degree, 168 nurses (33.9%) had a bachelor’s degree, and 243 nurses (49.1%) had a master’s degree or higher. Detailed demographic information is provided in Table 1.

**Table 1:** Demographic information of all participants.

Variables	Items	Number	Proportion (%)
Gender	Male	84	17.0
	Female	411	83.0

Age	18–30 years old	154	31.1
	31–45 years old	113	22.8
	46–60 years old	169	34.1
	Over 61 years old	59	11.9

Education background	Junior college	84	17.0
	Undergraduate college	168	33.9
	Master degree or above	243	49.1

Place of residence	Cities	336	67.9
	Countryside	159	32.1

Marriage	Married	292	59.0
	Unmarried	146	29.5
	Get divorced	41	8.3
	Widowed	16	3.2

Length of service	1–5 years	92	18.6
	6–10 years	76	15.4
	11–20 years	143	28.9
	More than 20 years	184	37.1

Type of job	Clinical nursing care	200	40.4
	Specialized nursing care	112	22.6
	Nursing management	134	27.1
	Nursing education	49	9.9

Training on digital literacy	Yes	342	69.1
	No	153	30.9

Duration of digital tools	≤ 2 h	49	9.9
	2–4 h	59	11.9
	4–6 h	163	32.9
	≥ 6 h	224	45.3

### 2.2. Research Instruments

#### 2.2.1. Digital Literacy Scale

The digital literacy scale was used to assess the digital literacy of clinical nurses. This scale, developed and validated by Avinç and Doğan [81], is a unidimensional scale consisting of 20 measurement items (e.g., “As a clinical nurse, do you frequently collaborate with other nurses using internet‐based software?”). The scale has been widely used in Chinese populations and demonstrates good cultural adaptability [82]. A five‐point Likert scale was used for scoring, ranging from “1 = *Strongly Disagree*” to “5 = *Strongly Agree*.” Higher scores indicate higher levels of digital literacy. In this study, the Cronbach’s alpha for the digital literacy scale was 0.961. Confirmatory factor analysis using AMOS 29.0 confirmed good structural validity (CMIN/DF = 3.985, GFI = 0.883, AGFI = 0.835, RMSEA = 0.078, CFI = 0.945, TLI = 0.929).

#### 2.2.2. Psychological Safety Climate Scale

The Psychological Safety Climate Scale was adapted from AC Edmondson [38], who developed the original Psychological Safety Scale for team‐level assessment. The scale consists of 7 measurement items (e.g., “As a clinical nurse, when a team member makes a mistake, they are not criticized”). A Chinese version of the scale, translated using the back‐translation method by Liu et al. [83], was used in this study. The five‐point Likert scale was applied, ranging from “1 = *Strongly Disagree*” to “5 = *Strongly Agree*.” Higher scores indicate higher levels of psychological safety climate. The Cronbach’s alpha for this scale was 0.849, and confirmatory factor analysis indicated good structural validity (CMIN/DF = 1.871, GFI = 0.986, AGFI = 0.971, RMSEA = 0.042, CFI = 0.989, TLI = 0.984).

#### 2.2.3. Situational Awareness Scale

The situational awareness scale was adapted from Schubert et al. [84]. To establish context, participants were instructed: “Imagine you are a nurse in the emergency department. Your surgical supervisor instructs you to suture a forearm laceration and then leaves the room. You have never performed this task before.” Following this scenario, participants answered 5 measurement items (e.g., “As a clinical nurse, do you agree that you will call a familiar general practitioner to assist you over the phone?”). This method has been widely used, as seen in the work of Hosseinpour and Keshmiri [85], to assess professional evaluations of medical scenarios. A five‐point Likert scale was used, ranging from “1 = Strongly Disagree” to “5 = Strongly Agree.” Higher scores indicate greater situational awareness. The Cronbach’s alpha for this scale was 0.811, and confirmatory factor analysis confirmed good structural validity (CMIN/DF = 1.686, GFI = 0.993, AGFI = 0.980, RMSEA = 0.037, CFI = 0.995, TLI = 0.990).

#### 2.2.4. Team Collaboration Ability Scale

The Nurse Collaboration Scale, which measures team collaboration ability, consists of four dimensions: conflict management, shared goals, communication and coordination, and professionalism and autonomy, comprising a total of 23 items. Developed by Liao et al. [86], this scale was validated using a Chinese population and demonstrates good cultural adaptability (e.g., “As a clinical nurse, when disagreements or conflicts arise, do everyone’s feelings and opinions get considered to achieve the best solution?”). A five‐point Likert scale was used, ranging from “1 = *Strongly Disagree*” to “5 = *Strongly Agree*.” Higher scores indicate greater team collaboration ability. The Cronbach’s alpha for this scale was 0.928, and confirmatory factor analysis confirmed good structural validity (CMIN/DF = 3.671, GFI = 0.880, AGFI = 0.830, RMSEA = 0.074, CFI = 0.933, TLI = 0.913).

### 2.3. Data Analysis

First, we used AMOS 29.0 to evaluate the goodness of fit for each variable and validate its construct validity. We employed SPSS 27.0 for reliability testing, normality analysis, descriptive statistics, correlation analysis, and common method bias testing. Subsequently, we applied the SPSS Process Model 6 to test the chain‐like mediation effects of psychological safety climate and situational awareness from a variable‐centered perspective. Third, we used Mplus to construct latent profile models of psychological safety climate and situational awareness, testing 1 to 5 profiles and selecting the optimal model based on fit indices. Finally, we conducted single‐factor analysis of variance (ANOVA) to examine significant differences in team collaboration ability across latent profile categories.

## 3. Results

### 3.1. Analysis of Common Method Bias

Common method bias can arise from the use of single measurement tools, consistent measurement environments, social expectations, and emotional states. To address this issue, we implemented anonymous data collection and avoided using guiding language in the questionnaires to minimize potential biases in responses. Simultaneously, we conducted Harman’s single‐factor test by performing exploratory factor analysis on all measured variables. The results showed that nine factors with eigenvalues greater than 1 were extracted, and the first factor explained 32.005% of the variance, which is below the critical threshold of 40%. This indicates that there is no common method bias in this study, supporting the validity of the data.

### 3.2. Descriptive Statistics and Correlation Analysis

We conducted correlation and descriptive tests on digital literacy, situational awareness, psychological safety climate, and team collaboration ability to examine the relationships between these variables, as shown in Table 2. According to RB Kline [87], the skewness of all variables in this study was less than ±3, and the kurtosis was less than ±8, indicating that the data followed a normal distribution. The correlation results revealed the following: digital literacy was significantly positively correlated with situational awareness (*r* = 0.433, *p* < 0.001, medium effect size per Cohen’s criteria), psychological safety climate (*r* = 0.463, *p* < 0.001, medium effect size), and team collaboration ability (*r* = 0.531, *p* < 0.001, large effect size). Situational awareness was also significantly positively correlated with psychological safety climate (*r* = 0.727, *p* < 0.001, large effect size) and team collaboration ability (*r* = 0.267, *p* < 0.001, small effect size). Additionally, psychological safety climate was significantly positively correlated with team collaboration ability (*r* = 0.174, *p* < 0.001, small effect size).

**Table 2:** Descriptive statistics and correlation analysis of each variable.

Variables	M	SD	Skewness	Kurtosis	1	2	3	4
1. Digital literacy	3.097	0.806	0.579	0.174	1			
2. Psychological safety climate	3.167	0.798	−0.147	0.250	0.463^∗∗∗^	1		
3. Situational awareness	3.095	0.849	−0.053	−0.162	0.433^∗∗∗^	0.727^∗∗∗^	1	
4. Team collaboration ability	3.037	0.757	0.810	0.509	0.531^∗∗∗^	0.267^∗∗∗^	0.174^∗∗∗^	1

^∗∗∗^ *p* < 0.001.

### 3.3. Variable‐Centered Analysis

Chain Mediation Test. We treated digital literacy as the independent variable, situational awareness and psychological safety climate as mediating variables, and team collaboration ability as the dependent variable. Using Process Model 6, we tested the chain mediation effects of situational awareness and psychological safety climate. The results showed that digital literacy had a significant associated with team collaboration ability (*β* = 0.504, 95% CI = [0.424, 0.584], *p* < 0.001); digital literacy also had significant associated with situational awareness (*β* = 0.130, 95% CI = [0.058, 0.202], *p* < 0.001) and psychological safety climate (*β* = 0.458, 95% CI = [0.380, 0.536], *p* < 0.001). Furthermore, psychological safety climate had a significant associated with situational awareness (*β* = 0.713, 95% CI = [0.641, 0.785], *p* < 0.001), and situational awareness had a significant associated with team collaboration ability (*β* = −0.137, 95% CI = [−0.235, −0.039], *p* < 0.001). Psychological safety climate also had a significant associated with team collaboration ability (*β* = 0.124, 95% CI = [0.017, 0.229], *p* < 0.05).

**Figure 3:**
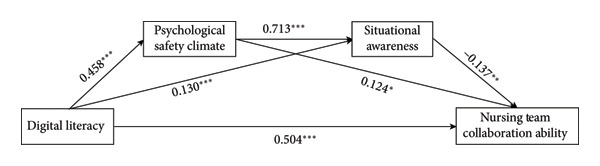
Graph of chain mediation path coefficients. ^∗∗∗^ *p* < 0.001^∗∗^ *p* < 0.01; ^∗^ *p* < 0.05.

Overall, situational awareness exhibited a significant partial mediating effect between digital literacy and team collaboration ability (*β* = −0.018, SE = 0.009, 95% CI = [−0.038, −0.004]); psychological safety climate also showed a significant partial mediating effect (*β* = 0.061, SE = 0.027, 95% CI = [0.008, 0.113]). Thus, both psychological safety climate and situational awareness had significant partial chain‐mediated effects on the relationship between digital literacy and team collaboration ability (*β* = −0.045, SE = 0.019, 95% CI = [−0.084, −0.011]). The regression coefficients and chain‐mediated pathway coefficients are detailed in Table 3, and the corresponding graphical representation is shown in Figure 3.

**Table 3:** Regression coefficient table of the chain‐type intermediary.

Regression equation		Overall fit index			Significance of regression coefficient		
Outcome variables	Predictive variables	*R*	*R* ^2^	*F*	*β*	*t*	95% CI
Psychological safety climate	Digital literacy	0.463	0.214	134.255^∗∗∗^	0.458	11.587^∗∗∗^	(0.380, 0.536)

Situational awareness	Psychological safety climate	0.735	0.540	288.901^∗∗∗^	0.713	19.414^∗∗∗^	(0.641, 0.785)
	Digital literacy				0.130	3.585^∗∗∗^	(0.058, 0.202)

Nursing team collaboration ability	Digital literacy	0.541	0.293	67.849^∗∗∗^	0.504	12.386^∗∗∗^	(0.424, 0.584)
	Psychological safety climate				0.124	2.294^∗^	(0.018, 0.229)
	Situational awareness				−0.137	−2.752^∗∗^	(−0.235, −0.039)

^∗^ *p* < 0.05.

^∗∗^ *p* < 0.01.

^∗∗∗^ *p* < 0.001.

Notably, the negative beta coefficient for situational awareness on team collaboration (*β* = −0.137) contrasts with the positive zero‐order correlation (*r* = 0.267, *p* < 0.001), suggesting a suppression effect. In mediation models, suppression occurs when the indirect effect has an opposite sign to the direct effect, inflating or revealing the total effect by accounting for opposing variance. Here, situational awareness appears to act as a suppressor, potentially due to its high correlation with psychological safety climate (*r* = 0.727), which may introduce inconsistent mediation where situational awareness captures negative residual variance after controlling for other predictors. This finding warrants further interpretation in the discussion.

This negative mediation coefficient indicates that, when psychological safety climate is included in the model, the unique relationship between situational awareness and team collaboration becomes negative. This suppression enhances the apparent strength of the positive direct effect of digital literacy on collaboration by removing irrelevant or opposing variance from situational awareness (e.g., aspects of hypervigilance that detract from teamwork). In practical terms, while situational awareness generally correlates positively with collaboration in bivariate analyses, its conditional effect turns negative once the shared positive variance with psychological safety climate is accounted for, highlighting a competitive or suppressive dynamic in the chain.

### 3.4. Individual Centered Analysis

#### 3.4.1. Latent Profile Analysis

To further examine the predictive effect of digital literacy on different types of nurses and the differences in psychological safety climate and situational awareness they exhibit, we used Mplus software to construct a 5‐class latent profile model to determine the best‐fitting model, as shown in Table 4. Specifically, we treated the individual measurement items of situational awareness and psychological safety climate as indicators and evaluated model fit. We determined a series of latent profile models ranging from two to five classes were compared using multiple statistical criteria, including the Akaike Information Criterion (AIC), Bayesian Information Criterion (BIC), sample‐size adjusted BIC (aBIC), the Lo–Mendell–Rubin likelihood ratio test (LMRT), the bootstrap likelihood ratio test (BLRT), and entropy, as well as theoretical interpretability and classification stability.

**Table 4:** Fit analysis of psychological safety climate and situational awareness.

Class	AIC	BIC	aBIC	Entropy	LMR (P)	BLRT (P)	Smallest proportion per class
1	18,102.378	18,203.287	18,127.111				
2	16,808.623	16,964.192	16,846.753	0.867	< 0.001	< 0.001	0.66/0.34
3	16,201.125	16,411.353	16,252.652	0.914	< 0.001	< 0.001	0.11/0.64/0.25
4	16,079.455	16,344.342	16,144.379	0.843	0.011	0.012	0.52/0.27/0.09/0.12
5	16,013.601	16,333.148	16,091.922	0.843	0.075	0.077	0.06/0.09/0.44/0.28/0.13

The results indicated that the entropy values for the 2‐ to 5‐latent profile models of perceived psychological climate and situational awareness all exceeded 0.80, suggesting that the classification accuracy of these models is acceptable. In the five‐class model, the *p* values for LMRT and BLRT were greater than 0.05, indicating that adding a fifth class did not significantly improve model fit compared to the 2‐ to 4‐class models. However, entropy, which measures the clarity of classification into latent profiles, is closer to 1, indicating clearer classification. Although the 4‐class model yielded a statistically significant LMRT (*p* = 0.011), the improvement in AIC and aBIC values relative to the 3‐class model was marginal, while entropy decreased markedly (0.914 ⟶ 0.843), suggesting a reduction in classification precision. Specifically, the 3‐class model exhibited lower BIC and aBIC values compared to the 4‐class model, which penalize model complexity and favor simpler structures to avoid overfitting [88]. Additionally, the 3‐class model yielded more substantively interpretable profiles (high, medium, and low levels of psychological safety climate and situational awareness), with balanced class sizes (11%, 64%, and 25%) that exceeded the recommended minimum of 5% per class, reducing the risk of unstable or artifactual small classes often seen in higher‐class solutions. In contrast, the 4‐class model introduced a small, potentially redundant class (< 5% of the sample), which lacked clear theoretical distinction from the existing profiles and could reflect sample‐specific noise rather than meaningful heterogeneity [89]. This decision aligns with best practices in LPA class enumeration, prioritizing models that balance statistical fit, parsimony, and practical interpretability in applied contexts like nursing psychology. Therefore, compared to the 2‐ and 4‐class models, the 3‐class latent profile model was considered the most parsimonious with the clearest classification (entropy = 0.914).

#### 3.4.2. Subgroup Classification

**Figure 4:**
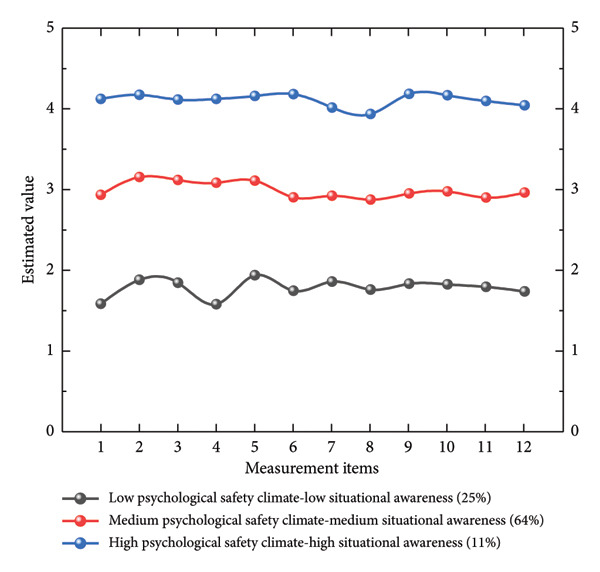
Proportion results of the three subgroups. Note. Measurement items 1–7 represent the psychological safety climate, while measurement items 8–12 represent situational awareness.

Based on the analysis in Table 4, we constructed three subgroups, as illustrated in Figure 4. The first class was labeled as high psychological safety climate‐high situational awareness, comprising 54 nurses and accounting for 11%. The second class was labeled as medium psychological safety climate‐medium situational awareness, comprising 317 nurses and accounting for 64%. The third class was labeled as low psychological safety climate‐low situational awareness, comprising 124 nurses and accounting for 25%.

#### 3.4.3. Latent Profiles and Nursing Team Collaboration Ability

We conducted a one‐way ANOVA to examine the differences in team collaboration ability across the three latent profiles, as shown in Table 5. The results indicated that the high situational awareness‐high psychological safety climate group (*M* = 3.244, SD = 0.706) had significantly higher team collaboration ability compared to the low situational awareness‐low psychological safety climate group (*M* = 2.621, SD = 0.717) and the medium situational awareness‐medium psychological safety climate group (*M* = 3.029, SD = 0.754), *p* < 0.001, *F* = 13.347, *η* ^2^ = 0.051, 95% CI = [0.018, 0.092]. These findings demonstrate significant differences in team collaboration ability across the three profiles, as detailed in Table 5.

**Table 5:** One‐way ANOVA for latent categories and nursing team collaboration ability.

Class	*N*	*M*	SD	*F*	*p*
1. High psychological safety climate—high situational awareness	55	3.244	0.706	13.347	< 0.001
2. Medium psychological safety climate—medium situational awareness	320	3.029	0.754		
3. Low psychological safety climate—low situational awareness	121	2.621	0.717		

## 4. Discussion

### 4.1. Personal‐Centered Perspective

This study employed latent profile analysis to identify three distinct latent profiles of situational awareness and psychological safety climate among clinical nurses. The first group, characterized by low psychological safety climate and a low situational awareness, exhibited sluggish responses to dynamic changes in the work environment and a pervasive fear of negative evaluations resulting from speaking up or making mistakes. The second group, marked by a medium psychological safety climate and situational awareness, demonstrated basic environmental interpretation skills, expressing concerns cautiously in most situations. The third group, characterized by a high psychological safety climate and situational awareness, demonstrated keen environmental awareness and the confidence to express opinions freely and take responsibility in a highly trusting atmosphere. These findings differ significantly from previous studies, which suggested that a high psychological safety climate leads to low situational awareness [90]. This discrepancy may stem from the unique characteristics of the healthcare industry, such as high risks, strong interdependence, and strict hierarchical systems [91, 92], which make the coexistence of high psychological safety and low situational awareness unsustainable among nurses. The vigilance of clinical nurses may either decrease due to suppression or gradually build up in supportive interactions, ultimately leading to a collaborative development model where situational awareness and psychological safety climate evolve together.

From the perspective of dynamic systems theory [93], situational awareness and psychological safety climate are not static traits but rather adaptive behavioral states that emerge in real‐time within specific nursing contexts. These states are the immediate expressions of the complex interplay between nurses’ internal cognitive‐emotional traits and external environmental pressures. For instance, in high‐pressure situations, nurses typically classified in the medium situational awareness and psychological safety climate group may shift to a high situational awareness state when facing a critically ill patient with rapidly deteriorating conditions. This highlights that the combination of high and low levels of situational awareness and psychological safety climate can provide a more comprehensive reflection of the psychological characteristics and behavioral patterns of clinical nurses. Specifically, the low situational awareness and low psychological safety climate state may indicate that nurses lack sensitivity to environmental information and the confidence to express their opinions in the work environment. In contrast, the high situational awareness and high psychological safety climate state reflect nurses’ ability to efficiently process environmental information and express their opinions openly and confidently within the team.

The distribution proportions of the latent profiles revealed the uneven existence of situational awareness and psychological safety climate states among clinical nurses. The high situational awareness and high psychological safety climate group accounted for the smallest proportion (11%), typically comprising experienced nurses in units with strong leadership support. The low situational awareness and low psychological safety climate group represented 25% of the sample and was often associated with individuals experiencing long‐term burnout or working in suppressive environments. The majority of nurses (64%) fell into the medium situational awareness and psychological safety climate group, forming the main body of clinical nurses. This distribution indicates that possessing high levels of both situational awareness and psychological safety is not common among nurses, but severely low‐performing states are also rare. Most nurses possess basic environmental interpretation skills and can express opinions when risks are controllable. Yet, they remain cautious about potential interpersonal risks, especially during cross‐hierarchical communication or when handling high‐uncertainty clinical decisions. This structure highlights the substantial potential for enhancing situational awareness and psychological safety climate among nurses, particularly in transitioning from medium to high levels of psychological safety and situational awareness.

Contemporary clinical nurses operate in an era dominated by digital information and social media, fostering a familiarity with immediate, transparent, and interactive communication patterns. However, the high uncertainty, strong hierarchical traditions, and ongoing digital transformation of healthcare environments shape their work realities. While abundant information sources could enhance situational awareness, information overload and fragmentation may lead to cognitive overload, weakening the ability to interpret critical situational cues. Additionally, the high‐stakes nature of healthcare, the prevalence of medical litigation, and persistent resource constraints collectively foster psychological insecurity. The intersection of these era‐specific and occupational characteristics renders nurses’ situational awareness highly susceptible to disturbances from information flows. At the same time, their psychological safety becomes fragile under authoritative communication patterns and performance pressures, easily slipping into medium or low situational awareness and psychological safety states, particularly in the absence of strong team leadership.

Given the significant impact of situational awareness and psychological safety climate on the work performance and professional development of clinical nurses, including their effects on nursing efficiency, team collaboration, and patient care quality, hospital administrators and nursing education institutions should prioritize addressing these issues. Specifically, healthcare organizations should establish supportive work environments, encourage nurses to express their opinions and suggestions, and provide psychological counseling resources to enhance the psychological safety climate. Additionally, nursing education departments should incorporate situational awareness training into nursing curricula to help nurses better perceive and respond to complex work environments. These measures could effectively improve clinical nurses’ situational awareness and psychological safety climate, thereby enhancing the efficiency of medical teams and the quality of patient care.

### 4.2. Variable‐Centered Perspective

#### 4.2.1. Theoretical Implications

This study validated the significant positive predictive effect of nurses’ digital literacy on nursing team collaboration capability. This finding underscores the importance of nurses’ ability to acquire, evaluate, and apply digital health information and tools in facilitating efficient collaboration during the digital transformation of healthcare. Nurses with high digital literacy can more accurately utilize digital tools to share critical patient data in real‐time, coordinate task handoffs via mobile nursing terminals, and engage in cross‐professional consultations via remote collaboration platforms. For instance, nurses skilled in using clinical decision support systems can quickly identify changes in patient risks and proactively initiate team alerts, reducing emergency response times [94]. This work efficiency, derived from digital capabilities, not only optimizes information flow but also minimizes communication friction and information errors [95]. This further strengthens the foundation of trust and the efficiency of team collaboration, providing a new pathway for high‐quality nursing services.

Improving nurses’ digital literacy reduces work aversion caused by information errors and enhances their sense of psychological safety within the team. Specifically, when nurses are proficient in digital tools, their technical anxiety decreases significantly, enabling them to ask questions and report errors in digital work scenarios without fear of negative evaluations. For example, nurses proficient in using medication safety systems are more likely to identify potential drug incompatibilities during team meetings. This digitally enabled safe expression allows teams to transform technical advantages into open knowledge sharing and constructive debates. Notably, digital literacy reduces the suppressive effects of hierarchical notions on communication by enhancing nurses’ control over technical interactions, making cross‐level collaboration more fluid, and increasing team collaboration efficiency.

In addition to psychological safety climate, situational awareness also plays a mediating role in the relationship between digital literacy and team collaboration capability. Nurses with high digital health literacy can more quickly and accurately identify changing clinical information when using EHRs, remote monitoring systems, or smart assistive devices, thereby forming a more comprehensive situational awareness. This cognitive advantage is further translated into critical capabilities in multitasking, team coordination, and rapid response, enhancing the timeliness and consistency of collaborative behaviors. Furthermore, nurses’ situational awareness helps strengthen their shared understanding of team goals, role distributions, and action plans, reducing information discrepancies and role conflicts in collaboration. Therefore, situational awareness not only serves as a cognitive bridge connecting digital literacy and collaboration capability but also provides cognitive support for nursing teams to maintain consistent operations in dynamic, high‐risk environments. However, the negative indirect effects through situational awareness (*β* = −0.018) and the chain pathway (*β* = −0.045) indicate suppression, where situational awareness opposes the positive direct effect of digital literacy on collaboration. This may reflect that excessive situational awareness, in high psychological safety contexts, leads to cognitive overload or hypervigilance, potentially hindering fluid team collaboration by increasing individual caution over collective action. To elaborate on the suppression effect, the inclusion of psychological safety climate in the model reveals a negative unique relationship between situational awareness and collaboration, despite their positive simple correlation. This occurs because psychological safety climate accounts for the shared positive variance, leaving situational awareness to explain residual negative variance—such as in scenarios where heightened awareness in safe teams leads to over‐analysis or decision paralysis, suppressing overall collaboration efficiency [96]. Similar suppression has been observed in nursing studies, for instance, where demoralization suppresses the relationship between stress and psychological well‐being in breast cancer patients [97]. This underscores the need for balanced interventions that mitigate potential downsides of situational awareness in supportive environments. Future studies should explore moderators like workload to clarify this inconsistent mediation. Nursing managers should promote situational awareness through simulations, multiscenario exercises, and the integration of digital tools to maximize the functional transformation of digital literacy in team collaboration [98].

#### 4.2.2. Practical Implications

Traditional nursing education often emphasizes the mastery of basic nursing techniques and disease knowledge. Still, this study underscores the importance of incorporating digital literacy as a crucial component in the professional competence framework for nurses. It is essential to note that merely teaching the technical skills of digital tools is insufficient; it is also necessary to combine situational awareness training with the cultivation of a psychological safety culture to truly transform digital capabilities into team performance. Therefore, nursing education systems should introduce scenario‐based digital interaction training, such as virtual reality‐based team simulations and task coordination operations using EHR systems, to enhance nurses’ environmental adaptability and technical sensitivity through highly realistic situational training. Additionally, psychological safety climate should be integrated into the educational process by fostering mutual trust, open feedback, and a culture of cooperation among students, laying the foundation for them to express opinions and collaborate efficiently in real clinical environments safely. This systemic nurturing approach not only helps improve nurses’ abilities in information identification, risk judgment, and collaborative communication but also effectively shortens the transition period from student to clinical backbone, aligning education with practice.

This study provides scientific evidence to support hospitals in optimizing their organizational management strategies and enhancing service quality. It clarifies the critical role of nurses’ digital literacy in enhancing collaboration capability and identifies the mediating mechanisms of psychological safety climate and situational awareness in organizational behavior regulation. Managers can adjust human resource management practices accordingly, incorporating digital capabilities and collaboration performance into recruitment, training, promotion, and performance evaluation standards. For instance, performance evaluations could include not only the accuracy of nursing operations but also the quality of information processing, efficiency, and team communication feedback on digital platforms. Additionally, fostering a supportive psychological climate is an important direction for management reform. Hospitals can establish psychological safety feedback mechanisms, anonymous suggestion platforms, and leader‐participatory management practices to encourage nurses to express their doubts and suggestions more freely during technical operations and clinical judgments, thereby enhancing transparency and collaboration. In summary, this study offers guidance for hospitals to cultivate a nursing organizational culture characterized by efficient collaboration, high situational responsiveness, and strong psychological safety, ultimately improving overall nursing quality and patient safety levels.

### 4.3. Limitations and Future Research Directions

Despite revealing the chain‐like mediating mechanism of psychological safety climate and situational awareness between nurses’ digital literacy and team collaboration capability, this study has certain limitations that may affect the external validity of the results. First, the study employed a cross‐sectional research design, with data collection concentrated in a specific time window, making it impossible to reveal the causal direction and long‐term dynamic changes between variables. Furthermore, while the mediation analysis using Process Model 6 suggests potential pathways through which digital literacy may be associated with team collaboration via situational awareness and psychological safety climate, causal inferences cannot be drawn due to the cross‐sectional nature of the data. This design precludes establishing temporal precedence or ruling out reverse causality (e.g., higher team collaboration might enhance digital literacy) and confounding factors, as highlighted in methodological literature on mediation in observational studies [99, 100]. The formation and evolution of digital literacy, situational awareness, and psychological safety climate are associated with various dynamic factors. Future studies should consider adopting longitudinal designs to dynamically observe how these psychological variables interact and impact team collaboration in digital environments.

Second, the sample was drawn from Grade A tertiary hospitals in eastern China. The regional concentration and relatively uniform organizational culture of the sample limit the generalizability of the findings to other regions, different types of healthcare institutions, and diverse nursing populations. Future research should expand the sample coverage to include hospitals of different levels, regions with significant cultural differences, and diverse nursing professionals to enhance the study’s representativeness and applicability.

Third, this study only examined psychological safety climate and situational awareness as mediating variables and did not investigate whether any moderating variables exist between them, which may lead to some bias in the interpretation of the results. For example, factors such as nurses’ age, clinical experience, technical training background, and the technical intensity of their departments may significantly influence their digital literacy and alter the roles of situational awareness and psychological safety climate. Additionally, the study did not fully consider the potential influence of organizational culture, leadership styles, and task complexity, which may play critical moderating roles in the mediating pathways. For instance, in authoritative or highly structured management environments, even nurses with high digital literacy may have their expressions and collaborative behaviors suppressed, preventing them from fully utilizing their potential.

Fourth, the reliance on self‐reported measures from a single source at one time point introduces potential limitations in the measurement approach and increases the risk of biases beyond common method bias, which we addressed using Harman’s single‐factor test. Specifically, social desirability bias may have influenced responses, as nurses could overreport favorable attributes such as high digital literacy or team collaboration to align with perceived professional norms [101]. Additionally, recall bias is a concern in this cross‐sectional design, where participants’ retrospective recollections of experiences (e.g., in situational awareness scenarios) might be inaccurate or incomplete due to memory distortions [102]. These biases could inflate correlations and affect the validity of our findings. To mitigate such issues in future research, incorporating multimethod approaches—such as objective behavioral observations, EHR data, or multirater assessments—would provide a more comprehensive and unbiased evaluation of the constructs.

To address these limitations, future research could employ mixed‐methods approaches, integrating qualitative interviews to explore contextual nuances, or experimental designs to test causal pathways. Moreover, cross‐cultural comparative studies would help validate the model’s applicability in diverse healthcare systems.

## 5. Conclusion

This study systematically explored how clinical nurses’ digital literacy enhances nursing team collaboration capability through a chain‐like mediating pathway involving situational awareness and psychological safety climate, adopting both variable‐centered and individual‐centered perspectives. The findings revealed the deep mechanisms by which nurses’ cognitive and emotional systems influence the efficiency of team collaboration in the digital era. The results confirm that nurses’ digital literacy not only significantly predicts their team collaboration capability but also transforms individual digital abilities into collaboration capabilities through enhanced psychological safety climate and situational awareness. Specifically, nurses with high digital literacy are better equipped to operate digital tools, process multisource data, and actively participate in decision‐making, reducing collaboration barriers caused by information errors or delays. Moreover, a high psychological safety climate encourages nurses to express opinions and provide feedback freely within the team, lowering communication costs and promoting the sharing of digital technology experiences. Furthermore, this sense of safety provides the emotional foundation for developing situational awareness, enabling nurses to more acutely perceive and understand dynamic clinical scenarios, thereby fostering efficient and precise team actions. The individual‐centered analysis revealed significant differences in team collaboration capabilities among nurses with varying combinations of situational awareness and psychological safety climate. The subgroup characterized by “high situational awareness‐high psychological safety climate” demonstrated the highest collaboration levels. This underscores the importance of optimizing situational awareness and psychological safety climate to enhance team collaboration capabilities among nurses. Overall, this study not only expands the theoretical boundaries of digital literacy in the nursing field but also provides evidence‐based recommendations for hospital administrators on nurse training, organizational culture development, and the optimization of collaboration mechanisms. Future research should explore the cross‐context applicability of this mechanism in multi‐level nursing collaboration systems and adopt longitudinal designs to capture the dynamic evolution of digital literacy’s influence, thereby driving the systematic upgrading and high‐quality development of digitalized nursing systems.

## Ethics Statement

I confirm that all methods were performed according to the relevant guidelines. All procedures were performed by the ethical standards outlined in the 1964 Declaration of Helsinki and its subsequent amendments. The study obtained informed consent from the all participants. This study involved human participants, and we obtained review and approval from the Academic Ethics Committee of Affiliated Hangzhou Hospital of Zhejiang Chinese Medical University (No. 2025KLL174).

## Consent

Please see the Ethics Statement.

## Conflicts of Interest

The authors declare no conflicts of interest.

## Author Contributions

Xiaoping Zhang and Zhongcheng An contributed to the methodology, data organization, and writing; Haifang Zhou contributed to the article’s data and paper writing; Bing Wu contributed to the article’s data; Lumeng Lu contributed to the methodology, data organization, and writing.

## Funding

This study was supported by Zhejiang Traditional Medicine and Technology Program, China (Grant No. 2025ZL056). Meanwhile, this study was supported by grants from the Construction Fund of Key Medical Disciplines of Hangzhou, China (Grant No. 2025HZPY06).

## Data Availability

The datasets used and/or analysed during the current study are available from the corresponding author on reasonable request.
